# An Underrecognized Cause of Recurrent Spontaneous Pneumothorax: Birt‐Hogg‐Dubé Syndrome With Isolated Pulmonary Manifestation

**DOI:** 10.1002/rcr2.70703

**Published:** 2026-09-08

**Authors:** Chia‐Ni Liu, Hsiao‐Chin Shen, Yi‐Chen Yeh

**Affiliations:** ^1^ Department of Chest Medicine Taipei Veterans General Hospital Taipei Taiwan; ^2^ Department of Medical Education Taipei Veterans General Hospital Taipei Taiwan; ^3^ School of Medicine National Yang Ming Chiao Tung University Taipei Taiwan; ^4^ Institute of Microbiology and Immunology National Yang Ming Chiao Tung University Taipei Taiwan; ^5^ Department of Pathology and Laboratory Medicine Taipei Veterans General Hospital Taipei Taiwan

**Keywords:** Birt‐Hogg‐Dubé syndrome, FLCN, folliculin, pneumothorax, pulmonary cysts

## Abstract

Birt‐Hogg‐Dubé (BHD) syndrome is a rare autosomal dominant disorder characterized by a classic triad of cutaneous fibrofolliculomas, bilateral pulmonary cysts and renal tumours. Pulmonary manifestations may occur in isolation, posing a significant diagnostic challenge. We report a 50‐year‐old non‐smoking woman with recurrent pneumothorax referred to our pulmonology clinic for dyspnoea on exertion. Chest CT revealed multiple bilateral pulmonary cysts with lower‐lobe predominance. Review of prior wedge resection specimens showed subpleural and parenchymal cysts lined by flattened epithelium. Negative immunohistochemical staining for HMB‐45 and GPNMB excluded lymphangioleiomyomatosis. Given the characteristic cyst pattern, germline whole‐exome sequencing identified a pathogenic heterozygous FLCN frameshift variant, confirming the diagnosis. Because of the lifetime risk of renal malignancy, early recognition is important and genetic testing is recommended when BHD is suspected.

## Graphical abstract

A 50‐year‐old woman presented with recurrent spontaneous pneumothorax and bilateral pulmonary cysts. Whole‐exome sequencing identified a pathogenic FLCN variant, confirming Birt‐Hogg‐Dubé (BHD) syndrome 15 years after symptom onset. This case highlights BHD as an underrecognized cause of diffuse cystic lung disease.

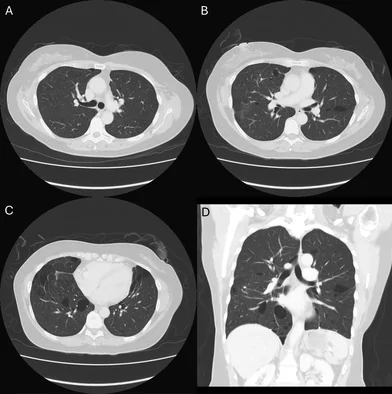

## 1 Introduction

Birt‐Hogg‐Dubé (BHD) syndrome is a rare autosomal dominant disorder caused by pathogenic germline variants in the FLCN gene, characterized by a triad of cutaneous fibrofolliculomas, bilateral pulmonary cysts and renal tumours. Its estimated prevalence ranges from 2 in 1,000,000 to 1 in 200,000, affecting males and females equally, although the true prevalence is likely underestimated [1]. Pulmonary manifestations are among the most prevalent features of BHD but may occur in isolation, without concurrent cutaneous or renal involvement [2]. This atypical presentation may lead to diagnostic delay. We report a case of BHD syndrome presenting exclusively with pulmonary manifestations in a non‐smoking woman.

## 2 Case Report

A 50‐year‐old non‐smoking woman with recurrent spontaneous pneumothorax was referred to our pulmonology clinic for intermittent dyspnoea on exertion. She first experienced pneumothorax 15 years ago and subsequently underwent wedge resections of the right upper lobe, right lower lobe and left upper lobe. Two months before this presentation, she experienced a further right‐sided pneumothorax managed at an outside facility.

On examination, her vital signs were within normal limits. Lung auscultation revealed no abnormal sounds. No skin papules were noted on her face, neck or upper trunk. Initial laboratory investigations, including complete blood count, renal function tests, liver enzymes, D‐dimer, erythrocyte sedimentation rate and C‐reactive protein, were all within normal limits. Autoimmune serologies, including rheumatoid factor, anti‐nuclear antibody, anti‐RNP, anti‐SSA, anti‐SSB, anti‐Scl‐70 and anti‐Jo‐1, were also unremarkable. Pulmonary function testing demonstrated an FEV1/FVC ratio of 0.89, FEV1 of 81% predicted, FVC of 79% predicted and TLC of 99% predicted. Chest radiography showed no residual pneumothorax but blunting of the left‐sided costophrenic angle. Abdominal ultrasonography showed no hepatic, pancreatic or renal abnormalities.

**FIGURE 1:**
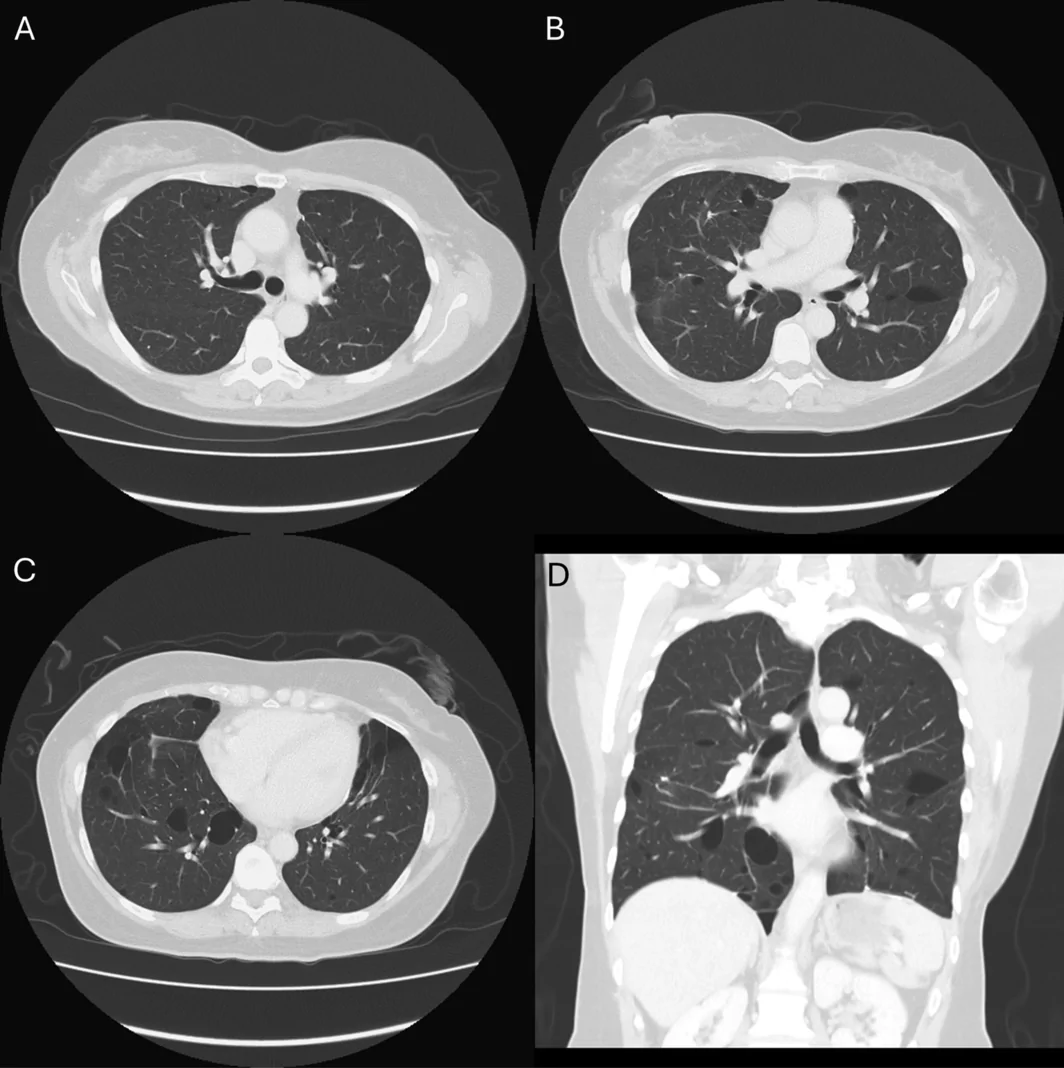
Axial chest CT images at different levels of the chest (A, upper; B, middle; C, lower) and coronal view (D) show multiple thin‐walled bilateral pulmonary cysts with medial and lower‐lobe predominance.

**FIGURE 2:**
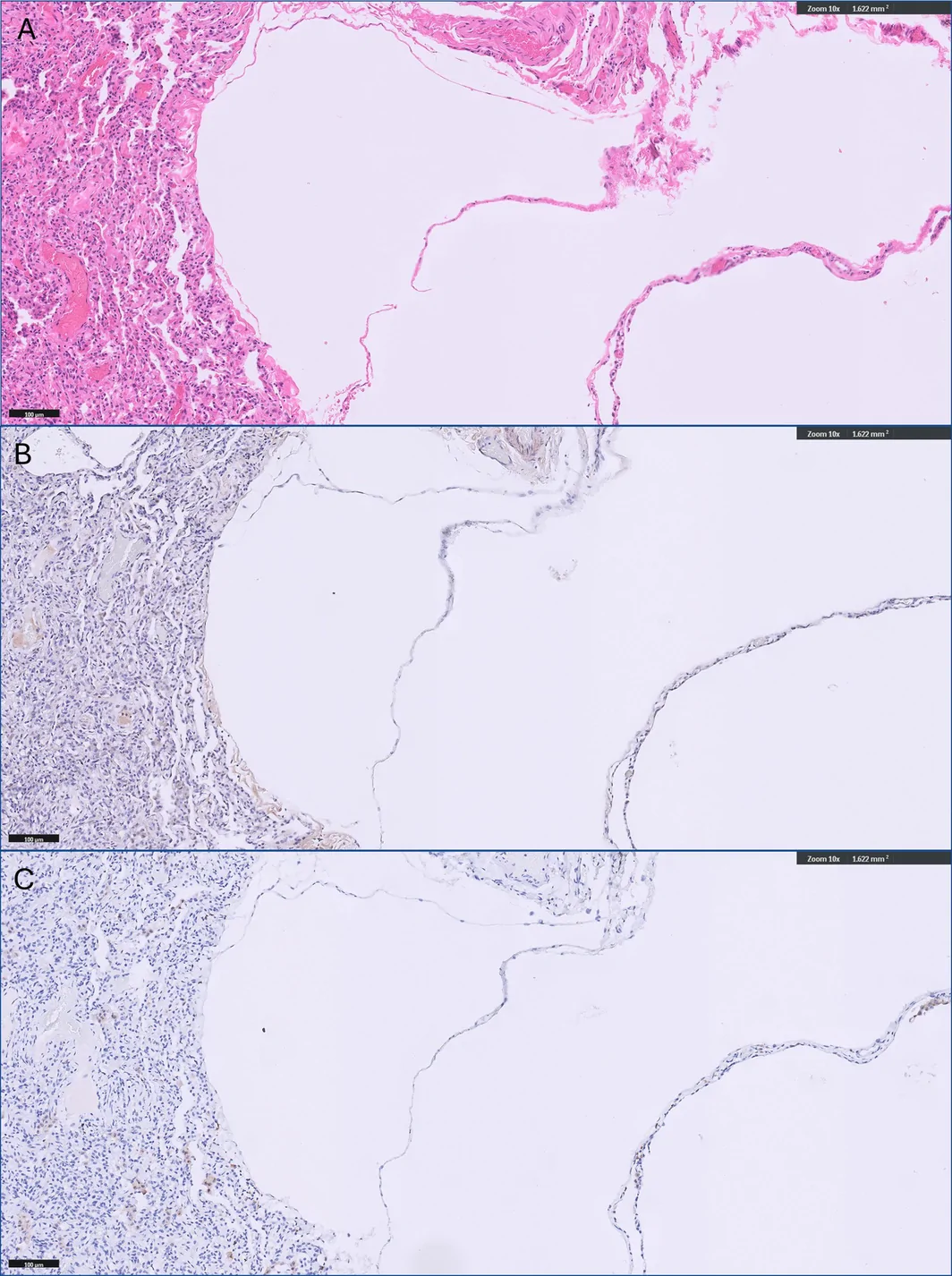
Haematoxylin–eosin staining of lung tissue from prior wedge resection demonstrating subpleural and parenchymal cystic spaces lined by flattened epithelium (A, scale bar = 100 μm). Immunohistochemical staining for HMB‐45 (B, scale bar = 100 μm) and GPNMB (C, scale bar = 100 μm) was negative.

Chest CT revealed multiple bilateral pulmonary cysts with predominance in the lower lobes and medial lung zones (Figure 1). Given the bilateral cystic lung disease, the differential diagnosis included lymphangioleiomyomatosis (LAM), pulmonary Langerhans cell histiocytosis (PLCH) and lymphoid interstitial pneumonia (LIP). The patient's non‐smoking status and unremarkable autoimmune serologies argued against PLCH and LIP, respectively. To evaluate for LAM, the prior wedge resection specimens were reviewed and demonstrated multiple subpleural and parenchymal cystic spaces lined by flattened epithelium, with an unremarkable non‐cystic parenchyma (Figure 2). Immunohistochemical staining for the LAM markers HMB‐45 and GPNMB was negative (Figure 2), excluding LAM. The combination of the lower‐lobe, medially predominant cyst pattern on CT and the absence of alternative diagnoses raised suspicion for BHD syndrome.

Based on clinical suspicion for BHD, germline whole‐exome sequencing was performed. A heterozygous frameshift variant in the FLCN gene (NM_144997.7:c.1579_1580insA; p.Arg527GlnfsTer75) was identified and classified as pathogenic per American College of Medical Genetics and Genomics guidelines, confirming the diagnosis of BHD syndrome. Formal dermatologic evaluation revealed no fibrofolliculomas. Abdominal imaging showed no renal tumours. Together, these findings established an isolated pulmonary presentation. The patient was counselled on the autosomal dominant inheritance pattern, and cascade genetic testing was offered to first‐degree family members. Annual renal surveillance with magnetic resonance imaging (MRI) was initiated given the lifetime risk of renal malignancy.

## 3 Discussion

This case illustrates that BHD syndrome may present with isolated pulmonary manifestations, without the classic dermatologic or renal features. Recognition of this presentation is critical, as the mean diagnostic delay from symptom onset has been reported at over 7 years [3]. BHD is caused by loss‐of‐function variants in FLCN, encoding folliculin, a tumour suppressor that regulates mTORC1 signalling. Loss of folliculin leads to dysregulated mTORC1 activity, implicated in alveolar cyst formation based on evidence from human BHD‐derived tissue and mouse models [4].

The classic clinical triad consists of fibrofolliculomas, pulmonary cysts and renal tumours. Spontaneous pneumothorax develops in approximately 30%–50% of patients at a median onset age of 30–38 years and recurs frequently [1]. BHD‐associated pulmonary cysts are characteristically thin‐walled and lentiform, with lower‐lobe and medial lung zone predominance [5]. The differential diagnosis for diffuse cystic lung disease includes LAM, PLCH and LIP [5]. LAM is the most important mimic and is distinguished by its diffuse distribution and positive HMB‐45 and GPNMB immunostaining. Diagnosis of BHD requires one major criterion (at least five fibrofolliculomas or a pathogenic FLCN variant) or two minor criteria (unexplained bilateral pulmonary cysts, early‐onset multifocal renal tumour before age 50 or a first‐degree relative with BHD) [2].

There is no approved disease‐modifying therapy. Acute pneumothorax is managed with standard approaches; surgical intervention or pleurodesis should be considered for recurrent episodes. Lifelong annual renal surveillance with MRI beginning at age 20 is recommended, as renal malignancy, though indolent, is the most serious complication [2]. Smoking should be strongly discouraged. Cascade genetic testing of first‐degree relatives is essential to identify asymptomatic carriers before clinical manifestations arise.

## Author Contributions

C.‐N.L. drafted the manuscript; Y.‐C.Y. provided pathologic evaluation and interpretation; H.‐C.S. critically reviewed and revised the manuscript. All authors approved the final version for submission.

## Funding

The authors have nothing to report.

## Ethics Statement

This study was approved by the Taipei Veterans General Hospital Institutional Review Board (IRB No. 2026‐05‐005BC).

## Consent

The authors declare that written informed consent was obtained for the publication of this manuscript and accompanying images using the consent form provided by the Journal.

## Conflicts of Interest

The authors declare no conflicts of interest.

## Data Availability

The data that support the findings of this study are available from the corresponding author upon reasonable request.
